# Chemokine CXCL13 in serum, CSF and blood–CSF barrier function: evidence of compartment restriction

**DOI:** 10.1186/s12987-020-0170-5

**Published:** 2020-02-24

**Authors:** Georg Pilz, Irma Sakic, Peter Wipfler, Jörg Kraus, Elisabeth Haschke-Becher, Wolfgang Hitzl, Eugen Trinka, Andrea Harrer

**Affiliations:** 1 Department of Neurology, Paracelsus Medical University, Ignaz-Harrer-Str 79, Salzburg, 5020 Austria; 2 Department of Laboratory Medicine, Paracelsus Medical University, Salzburg, Austria; 3 Departent of Neurology, Medical Faculty, Heinrich-Heine University of Düsseldorf, Düsseldorf, Germany; 4 Research Office, Biostatistics, Paracelsus Medical University, Salzburg, Austria; 5 Department of Ophthalmology and Optometry, Paracelsus Medical University, Salzburg, Austria; 6 Research Program Experimental Ophthalmology and Glaucoma Research, Paracelsus Medical University, Salzburg, Austria

**Keywords:** Chemokine, CXCL13, Cerebrospinal fluid, Blood–CSF-barrier, Inflammation, Biomarker, CSF/serum quotients

## Abstract

**Background and purpose:**

Elevation of the chemokine CXCL13 in CSF frequently occurs during active and acute CNS inflammatory processes and presumably is associated with B cell-related immune activation. Elevation levels, however, vary a lot and “leaking” of CXCL13 from blood across dysfunctional brain barriers is a possible source. The aim was to clarify the relation between CXCL13 concentrations in CSF, CXCL13 concentrations in serum and blood–CSF barrier (BCSFB) function for a correct interpretation of the intrathecal origin of CXCL13.

**Methods:**

We retrospectively analyzed CXCL13 of banked CSF/serum samples (n = 69) selected from patient records and categorized the CSF CXCL13 elevations as CXCL13 negative (< 30 pg/ml), low (30–100 pg/ml), medium (101–250 pg/ml), or high (> 250 pg/ml). CXCL13 concentrations in CSF and serum and the corresponding CSF/serum CXCL13 quotients (Qcxcl13) were compared to CSF/serum albumin quotients (QAlb) as a measure for BCSFB function. The CXCL13 negative category included two subgroups with normal and dysfunctional BCSFB.

**Results:**

Serum CXCL13 concentrations were similar across categories with median levels around 100 pg/ml but differed between individuals (29 to > 505 pg/ml). Despite clear evidence in serum, CXCL13 was detectable only at trace amounts (medians 3.5 and 7.5 pg/ml) in CSF of the two CXCL13 negative subgroups irrespective of a normal or pathological QAlb. Moreover, we found no association between CSF and serum CXCL13 levels or between QAlb and CSF CXCL13 levels in any of the CSF CXCL13-delineated categories. CXCL13 apparently does not “leak” from blood into CSF. This implies an intrathecal origin also for low CSF CXCL13 levels and a caveat for analyzing the Qcxcl13, because higher serum than CSF concentrations arithmetically depress the Qcxcl13 resulting in misleadingly low CSF/serum quotients.

**Conclusion:**

We demonstrated that CXCL13 does not cross from blood into CSF, not even during severe BCSFB dysfunction. CSF CXCL13 elevations therefore most likely always are CNS-derived, which highlights their relevance as indicator of inflammatory CNS processes. We recommend data should not be corrected for BCSFB permeability (QAlb) and not to calculate CSF/serum quotients for CXCL13 as these may introduce error.

## Introduction

The C-X-C motif ligand 13 (CXCL13) is a key homeostatic chemokine constitutively expressed in lymphoid organs and crucial for recruitment and compartmentalization of lymphocytes. Both B cells and follicular T helper cells, a special subgroup of CD4 T cells required for B cell activation, follow CXCL13 gradients towards B-T cell contact zones [1]. The primary sources of CXCL13 in lymph nodes are stromal cells and follicular dendritic cells [2, 3].

CXCL13, however, is not restricted to development and maintenance of lymphoid tissues but is also implicated in chronic inflammation via the formation of tertiary lymphoid structures (TLS) in a process called “ectopic lymphoneogenesis” [4]. TLS resemble germinal centers of B cell follicles but lack a stable structural organization. They develop at target organs of chronic inflammatory autoimmune and infectious diseases and in the surroundings of solid tumors [5–7].

CXCL13 also occurs in CNS inflammation and is the focus of biomarker research for Lyme neuroborreliosis (LNB), CNS lymphoma, and multiple sclerosis (MS) [8]. The broad spectrum of CNS inflammatory conditions with CXCL13 elevations in CSF also include bacterial/viral and aseptic meningitis, encephalitis, myelitis, and autoimmune encephalitis [9–14]. The function of CSF CXCL13 is only partly established and mainly attributed to B cell chemoattraction to the CNS [15]. The pathogenic relevance is associated with B cell-related immune activation during acute and active neuroinflammation [8]. It is proposed, that the CNS source for CXCL13 are monocytes in LNB, lesion infiltrating macrophages, perivascular stromal cells in primary CNS lymphoma, microglia or meningeal TLS cells in MS [15–17].

Peripheral blood is still another possible source, particularly in case of blood–CSF-barrier (BCSFB) dysfunction. Several studies use CSF/serum quotients of CXCL13 (Qcxcl13) and albumin (QAlb) for correcting for BCSFB permeability [18–20]. Observations from routine diagnostic data about negative CSF CXCL13 levels concurring with BCSFB dysfunction, however, made us challenge the assumption that CXCL13 crosses from blood to CSF as is established for albumin and immunoglobulins [21, 22]. We therefore investigated whether serum CXCL13 concentrations and BCSFB disruption contribute to CXCL13 elevations in CSF utilizing banked CSF/serum samples of patients of whom CSF CXCL13 concentrations and QAlb were available from records.

## Materials and methods

### Patient samples

This study included 69 samples collected 2017–2018 from patients assigned to lumbar puncture who gave informed consent (415-E/2286/7-2018) for inclusion of blood and CSF samples in the CSF Biobank Salzburg project, a collaboration of the Departments of Neurology and Laboratory Medicine. Sample processing and storage were in accordance to international standardization recommendations for CSF biomarker development [23].

Patient samples were specifically selected based on CXCL13 concentration in CSF, which we retrieved along with the CSF to serum albumin concentration quotient (QAlb) from records, and categorized as CSF CXCL13 negative (< 30 pg/ml), low (30–99 pg/ml), medium (100–250 pg/ml) and high (> 250 pg/ml). The cut-offs between categories were selected according to recommendations of Euroimmun CXCL13 ELISA instructions and published literature [24–27]. An age-dependent cut-off for normal QAlb was calculated as < (4 + age/15) × 10^−3^ [28].

The CXCL13 negative CSF category comprised two subgroups, with normal QAlb (< 30/N) and with pathological QAlb (< 30/P). The subgroup < 30/N of the CXCL13 negative category contained samples from patients with back pain, headache, dementia, stroke and nonspecific symptoms. The subgroup < 30/P of the CXCL13 negative category contained samples from patients with aseptic and purulent meningitis, spondylodiscitis, polyradiculitis, Wernicke encephalopathy, normal pressure hydrocephalus and cervical myelopathy. The CXCL13 low and medium categories contained samples from patients with aseptic and purulent meningitis, multiple sclerosis, facial paralysis, polyneuropathy, stroke and seizures. The CXCL13 high category contained samples from patients with Lyme neuroborreliosis, aseptic and purulent meningitis, and autoimmune myelitis and encephalitis.

The responsible ethics committee of the Country of Salzburg consented to this study (415-E/2430/3-2018).

### Cxcl13 elisa

CXCL13 concentrations of paired CSF and serum samples were analyzed by enzyme-linked immunosorbent assay (ELISA) according to the manufacturer’s recommendation (Euroimmun, Lübeck, Germany). This involved a 1:2 dilution of sera in a special serum-dilution buffer (provided in the kit). Since CSF was used undiluted in the assay, we multiplied the resultant serum concentration by factor 2. For quality control, we plotted CSF CXCL13 concentrations of the banked sample run against the corresponding diagnostic sample concentrations.

### Statistics

Data consistency was checked, and data were screened for outliers and normal distribution by using Kolmogorov–Smirnov test. Data distributions deviated from normal distributions. We used medians and interquartile ranges to summarize CXCL13 concentrations in CSF and serum, the Qcxcl13 and QAlb of the CSF CXCL13-delineated categories and subgroups, the Kruskal–Wallis ANOVA and Mann–Whitney U test for comparisons between categories (CXCL13 concentrations of CSF and sera, Qcxcl13, QAlb) and Wilcoxon’s rank sum test for comparing CSF versus serum CXCL13 concentrations within categories. For analyzing the associations of serum CXCL13 concentrations and QAlb with CSF CXCL13 concentrations regression analyses were done and Spearman correlations computed. Spearman’s rank-order correlation was used to test the relation of ELISA results between the diagnostic and banked CSF sample runs. All reported tests were two-sided, and p-values < 0.05 were considered as statistically significant. All statistical analyses and illustrations were done by use of SPSS Statistics 24.0 (IBM Germany GmbH), STATISTICA 13 (Hill, T. & Lewicki, P. Statistics: methods and applications. StatSoft, Tulsa, OK) and Microsoft Excel 2016 (Microsoft Office 2007, Redmond, USA).

## Results

The study sample included 22 CSF categorized as CXCL13 negative, 14 CSF categorized as CXCL13 low, 13 categorized as CXCL13 medium, and 20 categorized as CXCL13 high. The CXCL13 negative CSF category was divided into two subgroups, 10 samples with normal QAlb (< 30/N) and 12 samples with pathological QAlb (< 30/P). Table 1 summarizes median CXCL13 concentrations in CSF and serum, the resultant Qcxcl13 and the QAlb of the CSF CXCL13-delineated categories.

**Table 1:** Overview of CSF CXCL13-related categories

Groups	Categories		CSF CXCL13 (pg/ml)		NEG/N^Qalb^	Serum CXCL13 (pg/ml		NEG/N^Qalb^	Qcxcl13		NEG/N^Qalb^	QAlb		NEG/N^Qalb^
	CSF CXCL13	n	Median	IQR	p-value	Median	IQR	p-value	Median	IQR	p-value	Median	IQR	p-value
NEG/N^Qalb^	< 30	10	3.5	2–4.8		97	75–125		0.0	0.0–0.03		4.0	3.6–4.6	
NEG/P^Qalb^	< 30	12	7.5	3.3–20.5	ns	105	70–142	ns	0.1	0.0–0.2	ns	11.6	9.5–16.4	< 0.001
Low	30–99	14	61	41–77	< 0.01	98	52–186	ns	0.5	0.4–1.2	< 0.01	7.7	5.1–11.8	< 0.05
Medium	100–250	13	187	126–227	< 0.001	78	57–92	ns	2.6	1.4–3.2	< 0.001	11.7	4.6–16.2	< 0.001
High	> 250	20	> 505	378–505	< 0.001	113	76–272	ns	3.7	1.9–5.8	< 0.001	14.9	10.2–22.9	< 0.001

Significance testing and p-value refers to pair-wise comparisons with the NEG/N^QAlb^ category

*IQR* interquartile range, *ns*. not significant, *QAlb* albumin quotient, *Qcxcl13* CXCL13 quotient (c_(CXCL13)_CSF/c_(CXCL13)_Serum), *N*^*QAlb*^ refers to normal BCSFB function, *P*^*QAlb*^ refers to BCSFB dysfunction

**Fig. 1:**
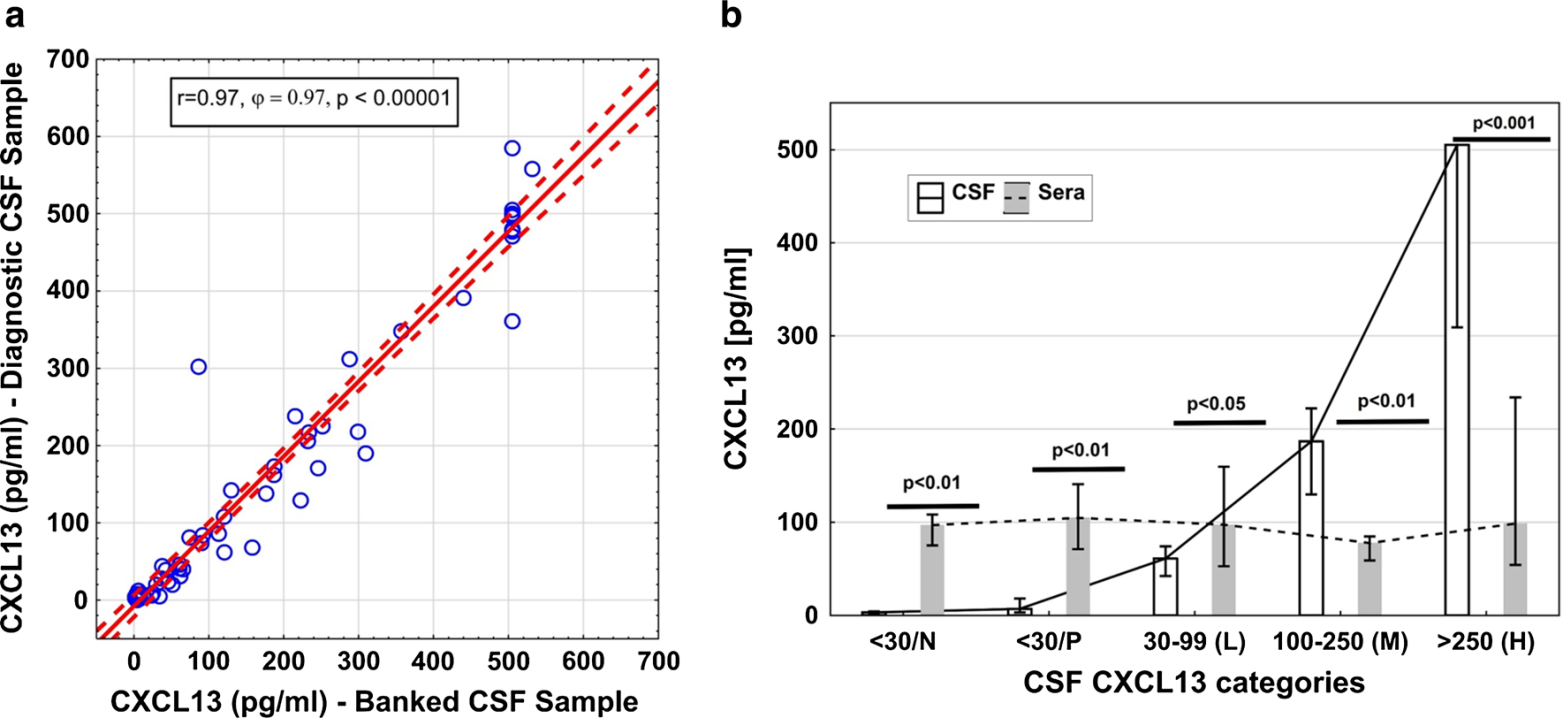
CXCL13 ELISA results. **a** Scatterplot showing the relation between CSF CXCL13 ELISA concentrations of the diagnostic and the banked samples. **b** Histogram illustrating the differences in CXCL13 concentrations between CSF (white) and blood (grey) across negative (< 30 pg/ml), low (L, 30–99 pg/ml), medium (M, 100–250 pg/ml) and high (H, > 250 pg/ml) categories and negative subgroups “< 30/N” (N, normal QAlb), “< 30/P” (P, pathological QAlb). Bars represent medians and IQR. Significance values indicate differences of CSF and serum CXCL13 concentrations within categories and subgroups. *IQR* interquartile range, *QAlb* albumin quotient

Importantly, we perfectly reproduced CXCL13 concentrations of the diagnostic run with the banked CSF samples (Spearman Rho 0.97; p < 0.001) (Fig. 1a). Median CSF CXCL13 levels of the CXCL13 negative subgroups were 3.5 (< 30/N) and 7.5 pg/ml (< 30/P), those of the CXCL13 low, medium and high categories were 61 pg/ml, 187 pg/ml and > 500 pg/ml, and thus consistent with preselection criteria of CXCL13 elevations (Table 1).

### Evidence of compartment restriction of CXCL13

In contrast to the differential CXCL13 concentrations in CSF, serum levels were rather uniform across categories with medians ranging from 78 to 113 pg/ml.

Median serum CXCL13 levels accordingly were higher than CSF levels of the negative (p < 0.01) and low (p < 0.05) categories. In addition, they were lower than CSF levels of the medium (p < 0.001) and high (p < 0.001) categories (Table 1, Fig. 1b).

To clarify whether and to what extent BCSFB dysfunction allowed CXCL13 “leak” from blood to CSF the two subgroups of the CXCL13 negative category described above, one with a normal BCSFB (< 30/N; median QAlb 4.0, IQR 3.6–4.6), the other with a significant BCSFB dysfunction (< 30/P; median QAlb 11.6, IQR 9.5–16.4; p = 0.001) were analyzed. Importantly, we detected only trace amounts of CXCL13 in CSF (medians 3.5 and 7.5 pg/ml), despite similar concentrations in serum (medians 97 and 105 pg/ml) and therefore independent of BCSFB function as measured by QAlb, (Table 1).

**Fig. 2:**
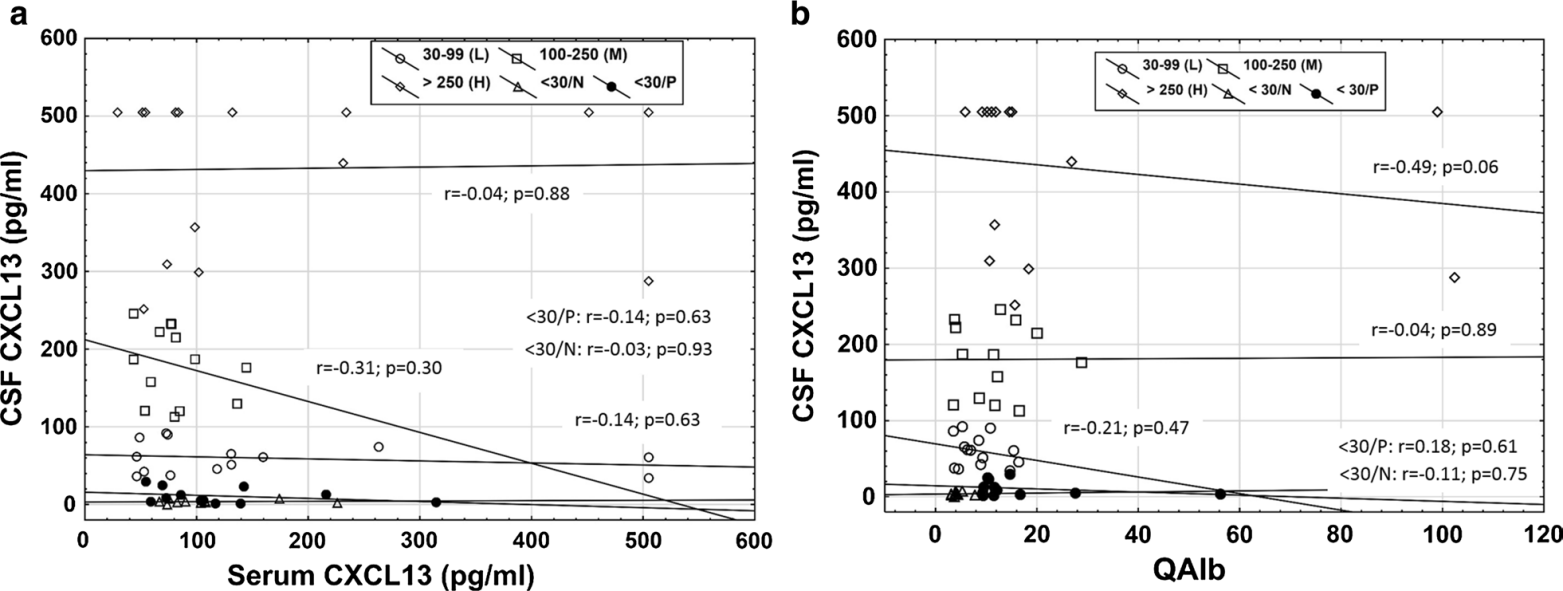
Categorized scatterplots with regression lines between **a** CSF CXCL13 and serum CXCL13 and **b** QAlb and CSF CXCL13 in the negative (< 30 pg/ml), low (30–99 pg/ml), medium (100–250 pg/ml) and high (> 250 pg/ml) categories of CSF CXCL13 elevations. *H* high, *L* low, *M* medium, *QAlb* albumin quotient, *< 30/N* negative CSF CXCL13/normal QAlb, *< 30/P* negative CSF CXCL13/pathological QAlb

Next, we investigated if there was any relation of CXCL13 levels in serum and CSF within each of the five subgroups but found no correlation (Fig. 2a). We also found no association between the degree of BCSFB dysfunction (QAlb) and CSF CXCL13 concentrations by plotting the QAlb against CXCL13 concentrations in CSF within each of the five subgroups (Fig. 2b).

Finally, and to further support the relevance of this finding, we compared BCSFB dysfunction (QAlb) between the subgroup < 30/P of the CXCL13 negative category and the three categories with low, medium and high CSF CXCL13 elevations. Expectedly, the QAlb of subgroup < 30/P (median 11.6, range 9.3–56.2) was higher than in the CXCL13 low category (30–99 (L); median 7.7, range 3.5–16.4; p < 0.05) but similar to the QAlb of the CXCL13 medium (100–250(M); median 11.7, range 3.5–28.8) and CXCL13 high (> 250 (H); median 14.9, range 5.9–102.4) categories.

Taken together, these data provide evidence for a compartment restriction because CXCL13 did not pass from blood to CSF even in case of BCSFB dysfunction.

### Implication of compartment restriction of CXCL13 on data interpretation

In the median and high CXCL13 categories, CSF levels exceeded serum levels with corresponding Qcxcl13 of median 2.6 and 3.8, clearly indicating an intrathecal origin of CXCL13. Whereas in the CXCL13 low category, serum levels exceeded CSF levels with a corresponding Qcxcl13 of median 0.5. Assuming diffusion dynamics across the BCSFB similar to albumin or immunoglobulins, a Qcxcl13 < 1, rather would suggest a blood-derived origin.

However, our data argued against CXCL13 passing from blood into CSF, which means that also low CSF levels most likely were intrathecally produced also in case of Qcxcl13 < 1.

Moreover, serum CXCL13 levels—although rather homogeneous across categories—strongly differed between individuals (range 29 to > 505 pg/ml). High serum concentrations arithmetically may depress the CSF/serum CXCL13 quotient and result in erroneously low Qcxcl13, as for instance very likely is the case in bacterial meningitis triggered by otitis media in Table 2.

**Table 2:** Discharge diagnosis and laboratory data of individual patients with serum CXCL13 concentrations > 250 pg/ml

	CXCL13 (pg/ml)		Qcxcl13	QAlb	CSF cells/µl
	CSF	Serum			
Lyme neuroborreliosis	> 505	> 505	na	16.5	80
Suspected primary CNS lymphoma	> 505	> 505	na	99.0	4
Bacterial meningitis triggered by otitis media	288	> 505	< 0.3	102.4	1275
*Spondylodiscitis*	*34*	> 505	< 0.05	*14.7*	115
Stroke in the context of arteritis temporalis	61	> 505	< 0.1	15.4	1
SLE with myelitis transversa	> 505	451	> 1.1	10.2	31
*Wernicke Encephalopathy and infection of unclear focus*	*3*	315	0.01	*16.7*	0
Lyme neuroborreliosis	> 505	284	> 1.8	24.6	112
HSV2 meningitis	74	263	0.3	8.5	140

Descending order as to serum CXCL13 concentrations. Cases with pathological QAlb but only traces of CXCL13 in CSF are written in italic letters

*na* not applicable, *QAlb* albumin quotient, *Qcxcl13* CXCL13 quotient (c_(CXCL13)_CSF/c_(CXCL13)_Serum), *SLE* systemic lupus erythematosus

### Serum CXCL13 elevations during neuroinflammation

To determine whether CXCL13 elevations in blood played a role or were otherwise related to CXCL13 elevations in CSF, we looked over serum CXCL13 data in more detail and retrieved diagnoses of those samples with serum CXCL13 concentrations > 250 pg/ml (Table 2).

Serum CXCL13 levels > 250 pg/ml occurred in nine patients (13%). Five of them had CXCL13 levels > 250 pg/ml in both serum and CSF. The associated diseases were LNB (n = 2), one suspected CNS lymphoma, one otitis media with bacterial meningitis and one SLE with myelitis transversa, all with evidence of both peripheral and CNS inflammatory foci. High serum but low levels in CSF (61 and 74 pg/ml) concurred with stroke in the context of arteritis temporalis and HSV2 meningitis.

The last two samples had high serum CXCL13 levels (315 and > 505 pg/ml) but borderline respectively negative CXCL13 levels in CSF (3 and 34 pg/ml), in the presence of significant BCSFB dysfunction (QAlbs of 16.7 and 14.7). The diagnoses were Spondylodiscitis, and Wernicke encephalopathy in combination with peripheral infection of unclear focus (italic letters, Table 2). Their inflammatory focus thus was peripheral and strongly indicated compartment restriction of blood from CSF CXCL13.

## Discussion

With this study, we provide evidence that CXCL13 does not enter CSF from blood, a finding of high relevance regarding the potential of CSF CXCL13 as diagnostic, prognostic, severity, and treatment response marker for neuroinflammatory conditions such as LNB, CNS lymphoma, demyelinating CNS diseases, and neuroinfections, and regarding a potential caveat when calculating CSF/serum CXCL13 quotients [8, 18–20, 29].

The fact that CXCL13 does not “diffuse” across the BCSFB similar to other serum proteins, is shown with our CXCL13 negative CSF category comprising of two subgroups with normal and dysfunctional BCSFB. Both subgroups had about median 100 pg/ml CXCL13 in serum, but only trace amounts of < 30 pg/ml in CSF irrespective of normal or compromised BCSFB function. CXCL13 elevations in CSF thus most likely are CNS-derived and signaling neuroinflammatory processes.

Referring to QAlb as measure for BCSFB permeability is best practice for estimating intrathecal production of immunoglobulins. This is important because immunoglobulins are capable of crossing the BCSFB, usually at low level. Albumin is strictly blood-derived and the QAlb a widely accepted measure for correcting an increased flux of blood-derived immunoglobulins into CSF during BCSFB disruption [22]. In case of pathogen-specific antibodies, an antibody index > 1.5 in favor of the CSF fraction serves as criterion for an intrathecal antibody production.

This approach cannot be applied to CXCL13 if CXCL13 does not similarly readily cross the BCSFB. Moreover, serum levels often exceed intrathecal levels of CXCL13 with the resultant Qcxcl13 < 1 misleadingly inferring lack of a CNS-derived fraction. We therefore recommend caution when correcting for BCSFB function and calculating the Qcxcl13 as these may introduce error, in particular in case of high serum versus low to intermediate CSF CXCL13 levels.

This brings us to serum CXCL13 levels, which—though similar across groups—differed pronouncedly between individuals. These results conform to current knowledge that CXCL13 normally is detectable in serum, but a normal range does not exist. Quantikine CXCL13 ELISA instructions (R&D Systems Europe, UK), for example, specified mean serum values of 81.9 pg/ml (range 39.4-252) derived from “apparently healthy donors without medical history”. Another study reported serum values of median 64 pg/ml (range 26–507) from 300 blood donors versus 94 pg/ml (range 28–417) in aseptic meningitis/facial palsy patients and 70 pg/ml (range 40–417) in patients with different neurological diagnoses [30]. These data are close to ours, although our ELISA was from a different provider.

The clinical relevance of serum CXCL13 in autoimmunity, infection, chronic inflammation and malignancy is widely apprehended and intensely investigated [31–35]. Every tenth of our neurology patients had serum elevations > 250 pg/ml. With LNB, systemic lupus with transverse myelitis, bacterial meningitis triggered by otitis media, and suspected CNS lymphoma we retrieved disease entities, which plausibly were associated with peripheral B-cell immune activity. Moreover, in LNB, CNS lymphoma, lupus, and bacterial meningitis patients CXCL13 was also highly elevated in CSF suggesting a concurrent CNS and peripheral inflammation. In sharp contrast, lack of CXCL13 in CSF despite severe BCSFB disruption in the spondylodiscitis and Wernicke encephalopathy patients indicated a sole peripheral focus of the CXCL13-associated inflammation.

Our finding that CXCL13 did not pass from blood into CSF, not even despite its small molecular size (~ 13 kDa) and not in cases of severe BCSFB dysfunction was somewhat unexpected. On the other hand, the role of CXCL13 as tissue-specific chemoattractant confined to lymph nodes and TLS, implicates the necessity for mechanisms restricting its diffusion and spatial distribution. The mechanisms for its tissue-specific local containment has recently been shown to rely on special molecular binding sites on CXCL13 for heparan sulfate side chains of proteoglycans on cells and within extracellular matrices [36]. These interactions are of utmost importance for chemokine gradients and haptotactic guidance of lymphocytes within tissues [37]. Whether proteoglycans and their side-chains also restrict the diffusion of chemokines across barriers is currently unresolved [38]. Given high serum levels concurring with severe BCSFB dysfunction and absence of CXCL13 in CSF, the concept of a molecular diffusion restriction appears highly plausible.

## Conclusion

Demonstrating that the chemokine CXCL13 does not cross from blood into CSF highlights its usefulness as biomarker for neuroinflammation, in particular, regarding conditions related to B cell activation and/or active humoral immune processes. Moreover, evidence that CSF CXCL13 is CNS-derived and independent from BCSFB integrity and serum levels requires cautious data interpretation with regard to calculating CSF/serum CXCL13 quotients and correcting for BCSFB permeability as these are prone to error.

In addition, we show that co-determination of serum CXCL13 has potential in allocating the focus of the inflammatory process, which may localize to the periphery or to the CNS or co-localize in both compartments with possible therapeutic consequences.

## Data Availability

The datasets analyzed during the current study are available from the corresponding author on request.

## Abbreviations

BCSFB: blood–CSF-barrier
CXCL13: C-X-C motif ligand 13
LNB: Lyme neuroborreliosis
MS: multiple sclerosis
QAlb: CSF/serum albumin quotients
Qcxcl13: CSF/serum quotients of CXCL13
TLS: tertiary lymphoid structures
